# Multiple mechanisms for enhanced plasmodesmata density in disparate subtypes of C_4_ grasses

**DOI:** 10.1093/jxb/erx456

**Published:** 2018-01-02

**Authors:** Florence R Danila, William Paul Quick, Rosemary G White, Steven Kelly, Susanne von Caemmerer, Robert T Furbank

**Affiliations:** 1 Research School of Biology, Australian National University, Canberra Australian Capital Territory, Australia; 2 ARC Centre of Excellence for Translational Photosynthesis, Australian National University, Canberra Australian Capital Territory, Australia; 3 International Rice Research Institute, Los Baños, Laguna, Philippines; 4 CSIRO Agriculture, Canberra Australian Capital Territory, Australia; 5 Department of Animal and Plant Sciences, University of Sheffield, Sheffield, UK; 6 Department of Plant Sciences, University of Oxford, Oxford, UK

**Keywords:** Bundle sheath, C_4_ decarboxylation types, C_4_ photosynthesis, grasses, mesophyll, pit field, plasmodesmata, symplastic transport

## Abstract

Proliferation of plasmodesmata (PD) connections between bundle sheath (BS) and mesophyll (M) cells has been proposed as a key step in the evolution of two-cell C_4_ photosynthesis; However, a lack of quantitative data has hampered further exploration and validation of this hypothesis. In this study, we quantified leaf anatomical traits associated with metabolite transport in 18 species of BEP and PACMAD grasses encompassing four origins of C_4_ photosynthesis and all three C_4_ subtypes (NADP-ME, NAD-ME, and PCK). We demonstrate that C_4_ leaves have greater PD density between M and BS cells than C_3_ leaves. We show that this greater PD density is achieved by increasing either the pit field (cluster of PD) area or the number of PD per pit field area. NAD-ME species had greater pit field area per M–BS interface than NADP-ME or PCK species. In contrast, NADP-ME and PCK species had lower pit field area with increased number of PD per pit field area than NAD-ME species. Overall, PD density per M–BS cell interface was greatest in NAD-ME species while PD density in PCK species exhibited the largest variability. Finally, the only other anatomical characteristic that clearly distinguished C_4_ from C_3_ species was their greater *S*_b_ value, the BS surface area to subtending leaf area ratio. In contrast, BS cell volume was comparable between the C_3_ and C_4_ grass species examined.

## Teaser

High plasmodesmata density in C_4_ grasses is a result of larger pit fields and/or more abundant plasmodesmata per pit field area.

## Introduction

Most plants obtain sugars by fixing atmospheric CO_2_ using the enzyme Rubisco (ribulose bis-phosphate carboxylase oxygenase). This process is inherently inefficient as O_2_ competes with CO_2_ at the enzyme’s active site, resulting in formation of compounds that cost energy to recycle in a process known as photorespiration. The first product of photosynthetic CO_2_ fixation by Rubisco is a three-carbon sugar, hence this process is known as C_3_ photosynthesis. Many tropical and sub-tropical plant lineages have independently evolved a more efficient photosynthetic biochemistry, termed C_4_ photosynthesis (Hatch, 1987; Sage *et al.*, 2011). Here, CO_2_ is first captured in mesophyll (M) cells as C_4_ acids, which then diffuse into bundle sheath (BS) cells, where Rubisco is located, and decarboxylated, resulting in greatly elevated local CO_2_ concentrations (Furbank and Hatch, 1987). This CO_2_-concentrating mechanism reduces photorespiration and enables Rubisco to operate close to its catalytic maximum (von Caemmerer and Furbank, 2003; Sage *et al.*, 2012). It is thought that a reduction in atmospheric CO_2_ concentration ~35 million years ago may have driven the evolution of this CO_2_-concentrating mechanism (Sage, 2004).

Although many plants conduct C_4_ photosynthesis, a range of anatomical and biochemical specialisations distinguish different C_4_ lineages. For example, subcategories of C_4_ plants are defined by the enzymes that catalyse the decarboxylation of the C_4_ acid: NADP malic enzyme (NADP-ME) type, NAD malic enzyme (NAD-ME) type, and phosphoenolpyruvate carboxykinase (PCK) type (Hatch, 1987; Furbank, 2011). Particularly in grasses, these biochemical differences are further elaborated by anatomical specialisations that include the presence of the mestome sheath between the BS and the vasculature in NAD-ME and PCK types but not in the NADP-ME type (Hattersley and Watson, 1976); suberisation of the BS cells in NADP-ME and PCK types but not in the NAD-ME type (Hattersley and Browning, 1981); and oval chloroplasts positioned centrifugally with mitochondria in BS cells of NADP-ME and most PCK types but elongated chloroplasts positioned centripetally with mitochondria in BS cells of the NAD-ME type (Hattersley and Watson, 1976; Hattersley and Browning, 1981; Dengler *et al.*, 1994; McKown and Dengler, 2007).

Over the last 35 million years, these evolutionary changes in anatomy and biochemistry arose independently at least 66 times (Sage *et al.*, 2012). In grasses, 22–24 distinct C_4_ lineages are found within the PACMAD (Panicoideae, Aristidoideae, Chloridoideae, Micrairoideae, Arundinoideae, and Danthonioideae) clade, specifically in the subfamilies Panicoideae, Aristidoideae, Chloridoideae, and Micrairoideae (GPWGII, 2012). These subfamilies comprise many highly productive crop species such as sugar cane, millets, and maize. On the other hand, the subfamilies Bambusoideae, Ehrhartoideae, and Pooideae, known as the BEP clade (GPWGII, 2012), contain no C_4_ species. These subfamilies include staple food grains such as rice, wheat, and barley. Demand for food crops is predicted to increase by at least 50% in the next 35 years (Hibberd *et al.*, 2008), and yield increases through traditional breeding of these C_3_ species will not meet this requirement. Recent breakthroughs in biotechnology may provide the opportunity to engineer the C_4_ photosynthetic pathway into C_3_ crops, which could potentially meet required improvements to feed the growing human population (Hibberd *et al.*, 2008).

The biochemistry of C_3_ and C_4_ photosynthesis has been well studied, with a strong focus on either down-regulation/knockout (von Caemmerer and Furbank, 2016) or overexpression (Kajala *et al.*, 2011) of one or more of the known key C_4_ photosynthetic enzymes in various plant systems to understand their function. Previous work has shown how leaf anatomical traits can be used to gain insight into C_4_ evolution (Hattersley and Watson, 1976; Dengler *et al.*, 1994; McKown and Dengler, 2007). These studies mainly investigated traits related to the specialised vascular anatomy of C_4_ plants known as Kranz anatomy: a wreath-like arrangement of BS and M cell layers enclosing the vascular bundles. This anatomical arrangement separates the biochemical CO_2_ ‘pump’ in the M from Rubisco in the BS, and provides a barrier to CO_2_ diffusion out of the BS compartment (von Caemmerer and Furbank, 2003; Sage *et al.*, 2012).

The requirement for metabolites to move at high rates between specialised C_4_ cell types has long been recognised to be important for C_4_ photosynthetic function (Osmond, 1971; Hatch and Osmond, 1976). Estimates of flux of photosynthetic metabolites between cell types in C_4_ leaves assume that the M–BS cell wall is impermeable due to the secondary thickening of cell walls between these cells (Danila *et al.*, 2016). Hence, these metabolites must move between cell types via plasmodesmata (PD), the symplastic nanochannels that span cell walls and provide both a cytoplasmic and an endoplasmic continuum for metabolite transport (Osmond and Smith, 1976; Robards, 1976; Overall and Blackman, 1996). In leaves, PD are distributed in groups called pit fields. The available data on PD distribution between leaf cells in C_3_ and C_4_ species show that C_4_ plants have a greater density of PD than C_3_ plants (Botha, 1992; Danila *et al.*, 2016). A major barrier to quantitatively examining these symplastic connections across diverse species has been the difficulty of the microscopy required to acquire statistically robust data (e.g. Botha, 1992). Recently, a high-throughput technique has been developed to assess PD density (Danila *et al.*, 2016). This technique combines high-resolution scanning electron microscopy (SEM), which allows analysis of individual PD within pit fields, and three-dimensional (3-D) immunolocalisation confocal microscopy for relatively rapid quantification of pit field distribution in a larger surface area across many cells within a leaf. Thus, it is now possible to quantify the PD connecting leaf cells of different C_4_ species and determine whether increased PD density at the M–BS interface is a conserved trait of C_4_ species and whether this density varies between different C_4_ subtypes. In this study, we quantify PD density between leaf cells in a selection of C_3_ and C_4_ grass species. These species encompass both BEP and PACMAD clades and include four origins of C_4_ photosynthesis comprising all C_4_ subtypes.

## Materials and methods

### Plant seeds and growth conditions

Seeds for *Astrebla lappacea*, *Leptochloa fusca*, *Panicum miliaceum*, *P. antidotale*, and *Urochloa panicoides* were gifted by Oula Ghannoum (Western Sydney University), *Brachypodium distachyon* seeds were obtained from CSIRO Black Mountain, and seeds from *Oryza sativa* cultivar Kitaake, *Hordeum vulgare* cultivar Yagan, *Triticum aestivum* cultivar Yecora 70, *P. bisulcatum*, *P. coloratum*, *Sorghum bicolor* cultivar Rooney, *Zea mays* cultivar B73, *Cenchrus ciliaris*, *Setaria viridis* cultivar A10, *Paspalum dilatatum*, *Chloris gayana*, and *P. maximum* (also known as *Megathyrsus maximus*) were obtained from the Research School of Biology (Australian National University). All seeds were germinated according to Danila *et al.* (2016). Growth conditions were maintained at 28 °C day/22 °C night temperatures, 60% relative humidity, 16 h light/8 h dark with peak at 1000 mmol quanta m^–2^ s^–1^ light intensity, and ambient CO_2_ concentration.

### Phylogenetic tree construction

To construct a phylogenetic tree for this analysis the predicted protein sequences for each of the 18 species were subject to orthogroup inference using OrthoFinder (Emms and Kelly, 2015) and a set of 60 single-copy orthogroups containing sequences from at least 16 of the 18 grass species were identified (Supplementary Dataset S1 at *JXB* online). These protein sequences were aligned using MergeAlign (Collingridge and Kelly, 2012), edited to remove all gap-containing columns, concatenated, and subjected to 1000 replicates of a non-parametric bootstrapped maximum-likelihood phylogenetic inference using FastTree (Price *et al.*, 2010). The full-length concatenated alignment was also used for Bayesian phylogenetic tree inference using mrBayes v 3.2.6 (Huelsenbeck and Ronquist, 2001). The amino acid model was set to JTT and the covarion was turned on. Two runs, each of four chains, were initiated and allowed to run for 100 000 generations sampling every 100 generations. Convergence was assessed through visual inspection of log-likelihood traces and through analysis of the standard deviation of split frequencies (σ^2^<0.00001).

### Leaf anatomical sample preparation

All leaf tissue preparations for light microscopy, SEM, and 3-D immunolocalisation confocal microscopy were as described by Danila *et al.* (2016). The middle portion of the youngest fully expanded leaf from three individual 9-d-old seedlings per species were collected and pooled. From this sample pool, leaf tissues were fixed and processed accordingly. For 3-D immunolocalisation confocal microscopy, leaf tissue was cleared using PEA-CLARITY (Palmer *et al.*, 2015), hybridised with β-1,3-glucan (callose) antibody, followed by Alexa488-tagged secondary antibody, and post-stained with calcofluor white to visualise cell walls (Danila *et al.*, 2016).

### Microscopy

Transverse sections of all grass leaves were imaged for light microscopy under 10× and 40× objectives using a Nikon Eclipse 50i upright microscope (Nikon Instruments). SEM was performed using a Zeiss Ultra Plus field emission scanning electron microscope at 3 kV. To quantify pit field distribution, two *z*-stacks from two leaf tissues per species were obtained using a Leica SP8 multiphoton confocal microscope (Leica Microsystems). Details can be found in Danila *et al.* (2016).

### Quantitative leaf anatomical measurement

Different from the conventional use of resin-embedded leaf tissue, BS cell area was measured from 25 to 50 individual cells of minor veins using virtual *z*-sections through entire confocal *z*-stacks for each species. BS cell volume was calculated by multiplying the BS cell area by BS cell length, which was measured using cell images (*n*=30 to 160) obtained from the paradermally orientated confocal micrographs of the same leaf *z*-stacks (Turrell, 1936). Vein diameter and interveinal distance (IVD) were measured using 10 to 25 individual minor veins from light micrographs of transverse leaf sections (see Supplementary Fig. S1). The bundle sheath surface area per unit leaf area, *S*_b_, was calculated using the equation described in Pengelly *et al.* (2010). To determine cell-to-cell PD connectivity among different subtypes of C_4_ photosynthesis, the frequency of PD within pit fields and density of pit fields per cell interface were analysed using SEM and confocal microscopy, respectively, in PCK (*n*=3 species), NAD-ME (*n*=4), and NADP-ME (*n*=6) grasses. For reference, representative grasses from C_3_ BEP (*n*=4) and C_3_ PACMAD (*n*=1) were also measured. Quantitation of PD per µm^2^ pit field (*n*=18 or more whole pit fields obtained from SEM), percent pit field per cell interface area (*n*=5 or more maximum intensity projection images generated from two confocal *z*-stacks), and PD per µm^2^ cell interface were carried out as described in Danila *et al.* (2016). PD quantification values used for *O. sativa*, *T. aestivum*, *Z. mays*, and *S. viridis* were as reported in Danila *et al.* (2016) (see Supplementary Table S1 for specific details). The cross-sectional area of at least 40 individual PD enclosed by the wall collar (Faulkner *et al.*, 2008) (termed as PD area here) located in the M–BS cell interface was measured from SEM images. PD area per M–BS interface area and PD area per unit leaf area were calculated as follows:

$$\text{PD area per M}–\text{BS interface area}=\text{PD area}\times \text{PD per }µ{\text{m}}^{2}\text{ M}–\text{BS cell interface}$$

$$\text{PD area per unit leaf area}=\text{PD area per M}–\text{BS interface area}\times S_{\text{b}}$$

All anatomical measurements were performed using ImageJ software (https://imagej.nih.gov/ij/).

### Statistical analysis

Statistical analyses were carried out using one-way (photosynthetic type and species) ANOVA (OriginPro 9.1, OriginLab Corporation). Means were grouped using a *post hoc* Tukey test.

## Results

### C_4_ origin and lineage representation

**Fig. 1.**
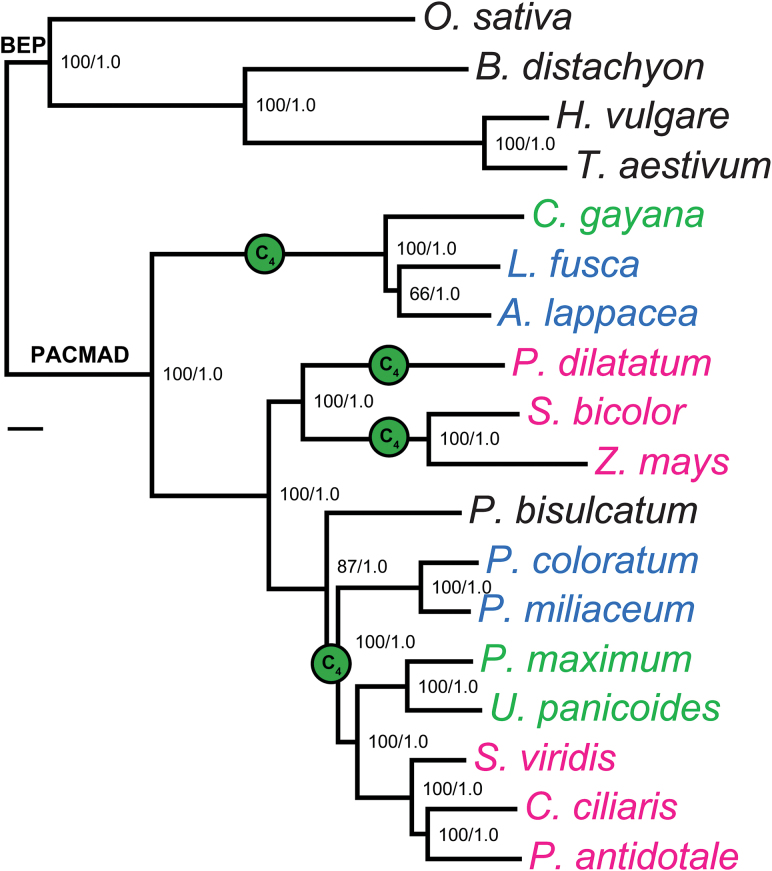
Phylogenetic tree of the C_3_ and C_4_ grass species examined in this study, which is identical to the expected topology from GPWGII (2012). Species names are colour-coded according to photosynthetic types: black, C_3_; green, C_4_ PCK; blue, C_4_ NAD-ME; magenta, C_4_ NADP-ME. The four independent evolutionary origins of C_4_ photosynthesis are indicated with green circles at the midpoint of the branches. Support values at internal nodes are ML/B, where ML is the percentage of non-parametric bootstrap replicates that support the bipartition, and B is the Bayesian posterior probability for the bipartition. The scale bar indicates 0.1 substitutions per site.

A phylogenetic tree of the 18 species was adapted from GPWGII (2012). It is currently thought that this set of species encompass four independent origins of C_4_ photosynthesis (GPWGII, 2012). The independent evolutionary origins of C_4_ are indicated in Fig. 1 where species are colour-coded according to their photosynthetic type (Table 1); this coding and species order are retained throughout the paper.

**Table 1.** Photosynthetic type, taxonomic group (subfamily and tribe), and C_4_ lineage representation of the 18 grass species examined

Grass species	Photosynthetic type	Subfamily	Tribe	C_4_ lineage*
*Oryza sativa* cv Kitaake	C_3_, BEP	Ehrhartoideae	Oryzeae	Not applicable
*Brachypodium distachyon*	C_3_, BEP	Pooideae	Brachypoideae	Not applicable
*Hordeum vulgare* cv Yagan	C_3_, BEP	Pooideae	Triticeae	Not applicable
*Triticum aestivum* cv Yecora 70	C_3_, BEP	Pooideae	Triticeae	Not applicable
*Chloris gayana*	C_4_ PCK, PACMAD	Chloridoideae	Cynodonteae	Chloridoideae
*Leptochloa fusca*	C_4_ NAD-ME, PACMAD	Chloridoideae	Cynodonteae	Chloridoideae
*Astrebla lappacea*	C_4_ NAD-ME, PACMAD	Chloridoideae	Cynodonteae	Chloridoideae
*Paspalum dilatatum*	C_4_ NADP-ME, PACMAD	Panicoideae	Paspaleae	*Paspalum*
*Sorghum bicolor* cv Rooney	C_4_ NADP-ME, PACMAD	Panicoideae	Andropogoneae	Andropogoneae
*Zea mays* cv B73	C_4_ NADP-ME, PACMAD	Panicoideae	Andropogoneae	Andropogoneae
*Panicum bisulcatum*	C_3_, PACMAD	Panicoideae	Paniceae	C_3_ sister to MPC
*Panicum coloratum*	C_4_ NAD-ME, PACMAD	Panicoideae	Paniceae	MPC
*Panicum miliaceum*	C_4_ NAD-ME, PACMAD	Panicoideae	Paniceae	MPC
*Panicum maximum*	C_4_ PCK, PACMAD	Panicoideae	Paniceae	MPC
*Urochloa panicoides*	C_4_ PCK, PACMAD	Panicoideae	Paniceae	MPC
*Setaria viridis* cv A10	C_4_ NADP-ME, PACMAD	Panicoideae	Paniceae	MPC
*Cenchrus ciliaris*	C_4_ NADP-ME, PACMAD	Panicoideae	Paniceae	MPC
*Panicum antidotale*	C_4_ NADP-ME, PACMAD	Panicoideae	Paniceae	MPC

*According to GPWGII, (2012).

MPC, Melinidinae, Panicinae, and Cenchrinae.

### Plasmodesmata in C_4_ grasses

**Fig. 2.**
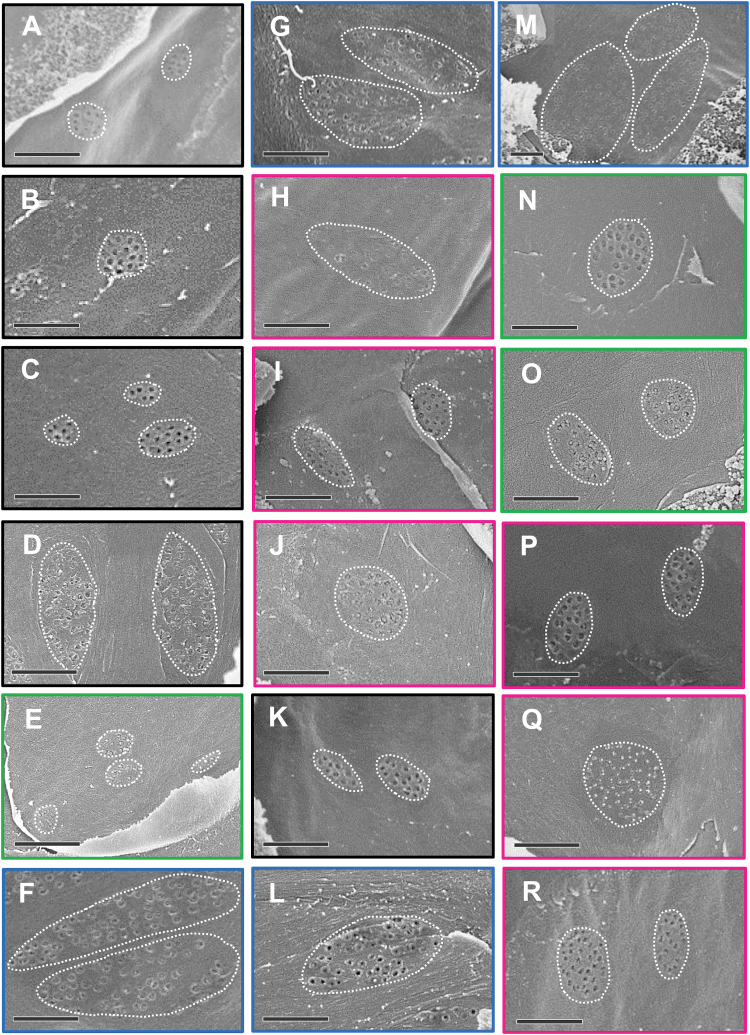
Scanning electron micrographs showing pit field size on the mesophyll–bundle sheath cell interface of C_3_ and C_4_ grass leaves. (A–D) C_3_ BEP species (black frames): (A) *Oryza sativa*, (B) *Brachypodium distachyon*, (C) *Hordeum vulgare*, and (D) *Triticum aestivum*. (E, N, O) C_4_ PCK (green frames): (E) *Chloris gayana*, (N) *Panicum maximum*, and (O) *Urochloa panicoides*. (F, G, L, M) C_4_ NAD-ME (blue frames): (F) *Leptochloa fusca*, (G) *Astrebla lappacea*, (L) *Panicum coloratum*, and (M) *Panicum miliaceum*. (H–J, P–R) C_4_ NADP-ME (magenta frames): (H) *Paspalum dilatatum*, (I) *Sorghum bicolor*, (J) *Zea mays*, (P) *Setaria viridis*, (Q) *Cenchrus ciliaris*, and (R) *Panicum antidotale*. (K) C_3_ PACMAD (black frame): *Panicum bisulcatum*. Each pit field is enclosed with a dotted white line. The micrograph of *P. miliaceum* (M) is zoomed out to show whole pit fields. Scale bars are 1 µm.

**Fig. 3.**
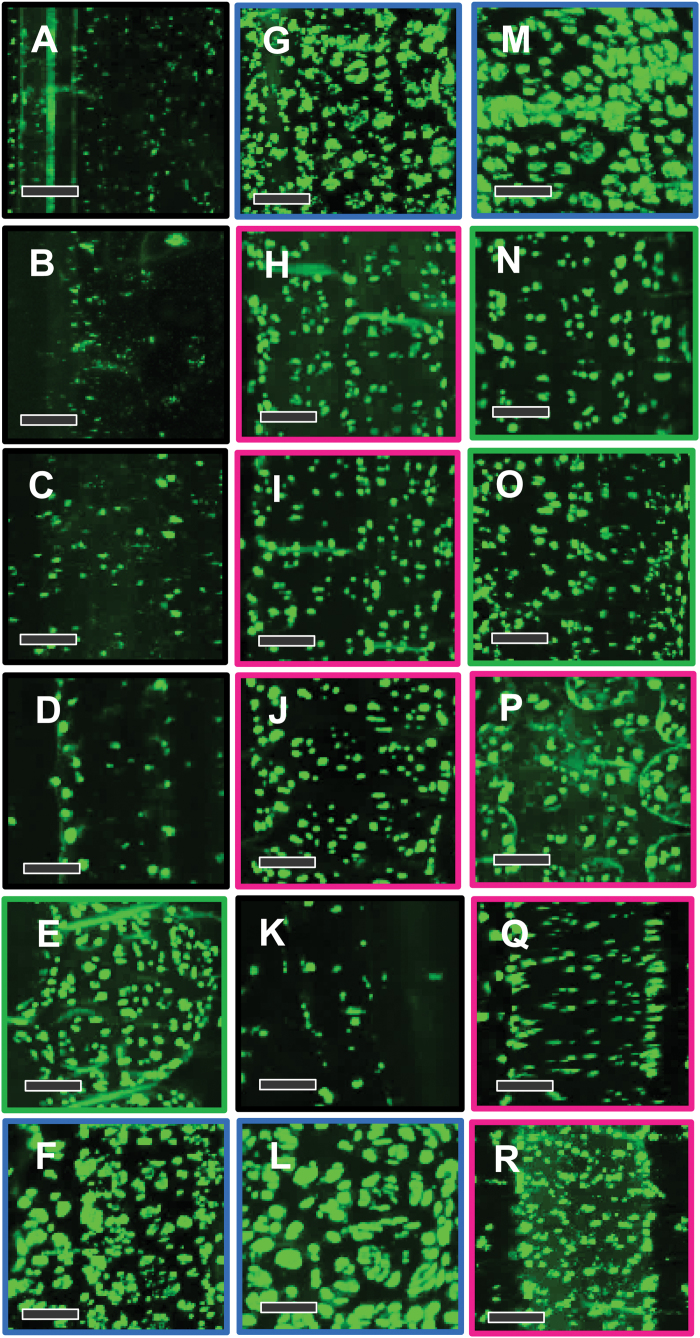
Confocal micrographs showing the patterns of pit field distribution on the mesophyll–bundle sheath cell interface of C_3_ and C_4_ grass leaves. (A–D) C_3_ BEP species (black frames): (A) *Oryza sativa*, (B) *Brachypodium distachyon*, (C) *Hordeum vulgare*, and (D) *Triticum aestivum*. (E, N, O) C_4_ PCK (green frames): (E) *Chloris gayana*, (N) *Panicum maximum*, and (O) *Urochloa panicoides*. (F, G, L, M) C_4_ NAD-ME (blue frames): (F) *Leptochloa fusca*, (G) *Astrebla lappacea*, (L) *Panicum coloratum*, and (M) *Panicum miliaceum*. (H–J, P–R) C_4_ NADP-ME (magenta frames): (H) *Paspalum dilatatum*, (I) *Sorghum bicolor*, (J) *Zea mays*, (P) *Setaria viridis*, (Q) *Cenchrus ciliaris*, and (R) *Panicum antidotale*. (K) C_3_ PACMAD (black frame): *Panicum bisulcatum*. Green fluorescence corresponds to pit fields. Scale bars are 10 µm.

**Fig. 4.**
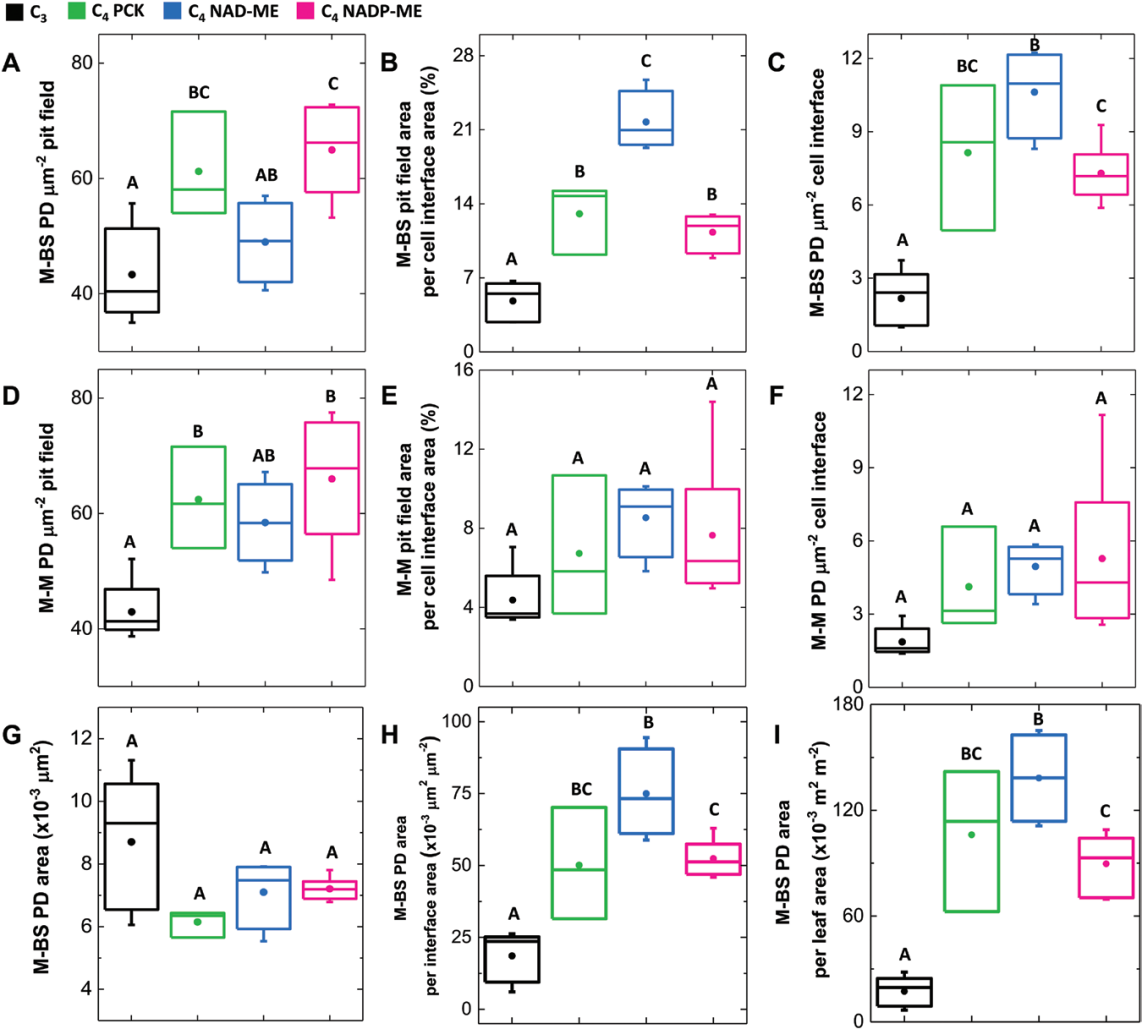
Distribution of plasmodesmata trait values among photosynthetic types. The distribution of nine variables is summarised by boxplots individually for the C_3_ BEP and PACMAD (black, *n*=5), C_4_ PCK (green, *n*=3), C_4_ NAD-ME (blue, *n*=4), and C_4_ NADP-ME (magenta, *n*=6). Box and whiskers represent the 25 to 75 percentiles, and the minimum and maximum distribution. Means are denoted by dots. Letters show the statistical ranking using a *post hoc* Tukey test among photosynthetic types (different letters indicate differences at *P*<0.05). Data for individual species are given in Supplementary Table S1. M, mesophyll; BS, bundle sheath; PD, plasmodesmata.

Analysis of pit field size (Fig. 2, Supplementary Table S1) and patterns of pit field distribution (Fig. 3) revealed that NAD-ME species had the largest and most abundant pit fields (in terms of area coverage) on the M–BS cell interface. Both NADP-ME and PCK species had smaller and less abundant pit fields. C_3_ species, from both the BEP (Figs 2A–D, 3A–D) and PACMAD (Figs 2K, 3K) clades, had considerably less abundant, smaller pit fields. The large pit fields in NAD-ME species had more widely spaced PD (Fig. 2, Supplementary Table S1). Indeed, NAD-ME species had fewer PD per pit field area on the M–BS cell interface compared to NADP-ME and most PCK species (Fig. 4A, Supplementary Table S1). This was offset by the greater percent pit field area per M–BS cell interface area in NAD-ME species compared to NADP-ME and PCK species (Fig. 4B, Supplementary Table S1). The resulting PD density per M–BS cell interface was greater in NAD-ME compared to NADP-ME species, with large variation observed amongst the PCK species (Fig. 4C, Supplementary Table S1). C_4_ species also had greater PD density on the M–M cell interface relative to the C_3_ species but there was no substantial variation among the C_4_ subtypes (Figs. 4D–F, Supplementary Table S1). Estimates of the cross-sectional area of individual PD revealed no significant differences between the two photosynthetic pathways and among decarboxylation types (Fig. 4G, Supplementary Table S1). The proportion of the M–BS cell interface populated by PD (Fig. 4H, Supplementary Table S1) and the M–BS PD area per unit leaf area (Fig. 4I, Supplementary Table S1) were greater in C_4_ species than C_3_ species, and followed the pattern of PD density for the C_4_ decarboxylation types.

### Bundle sheath of C_4_ grasses

**Fig. 5.**
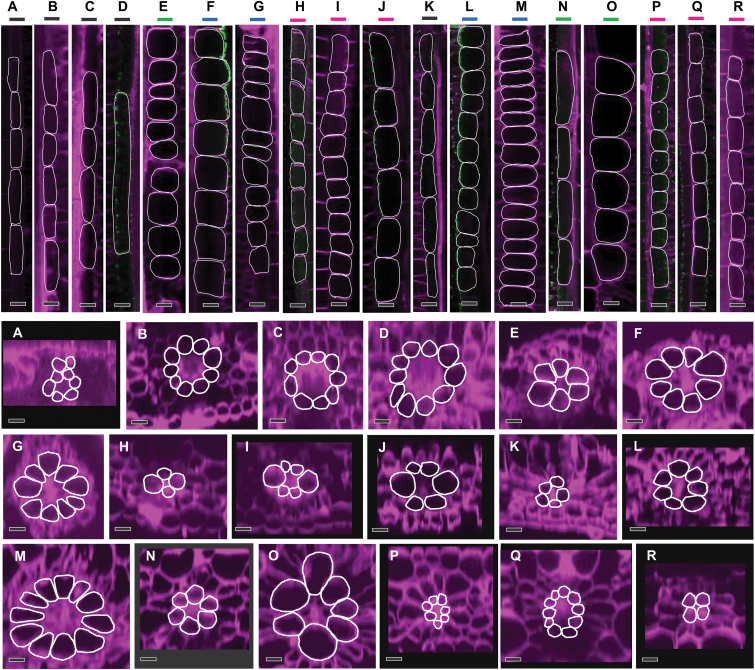
Confocal micrographs obtained from 3-D stacks of C_3_ and C_4_ grass leaves showing bundle sheath cells in paradermal (top) and transverse (bottom) orientations. (A–D) C_3_ BEP species (black lines): (A) *Oryza sativa*, (B) *Brachypodium distachyon*, (C) *Hordeum vulgare*, and (D) *Triticum aestivum*. (E, N, O) C_4_ PCK (green lines): (E) *Chloris gayana*, (N) *Panicum maximum*, and (O) *Urochloa panicoides*. (F, G, L, M) C_4_ NAD-ME (blue lines): (F) *Leptochloa fusca*, (G) *Astrebla lappacea*, (L) *Panicum coloratum*, and (M) *Panicum miliaceum*. (H–J, P–R) C_4_ NADP-ME (magenta lines): (H) *Paspalum dilatatum*, (I) *Sorghum bicolor*, (J) *Zea mays*, (P) *Setaria viridis*, (Q) *Cenchrus ciliaris*, and (R) *Panicum antidotale*. (K) C_3_ PACMAD (black line): *Panicum bisulcatum*. Bundle sheath cells are outlined in white. Scale bars are 20 µm.

**Fig. 6.**
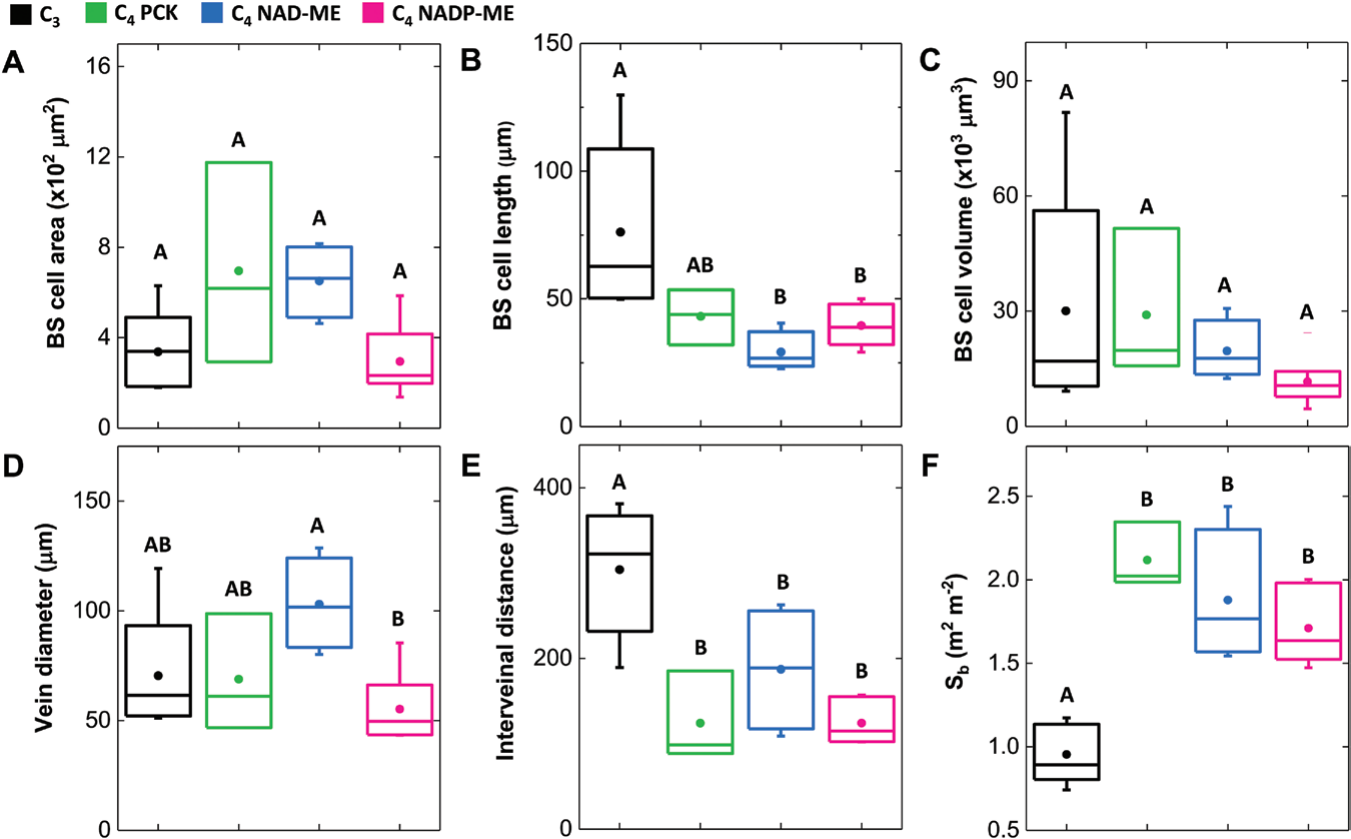
Distribution of bundle sheath-related trait values among photosynthetic types. The distribution of six variables is summarised by boxplots individually for the C_3_ BEP and PACMAD (black, *n*=5), C_4_ PCK (green, *n*=3), C_4_ NAD-ME (blue, *n*=4), and C_4_ NADP-ME (magenta, *n*=6). Boxes and whisker plots are as described in Fig. 4. Data for individual species are given in Supplementary Table S2. BS, bundle sheath; *S*_b_, bundle sheath surface area per unit leaf area.

Our 3-D approach to measure BS cell cross-sectional areas and volumes used confocal micrographs derived from *z*-stacks of the leaf (Fig. 5). Measurement of the BS cell cross-sectional areas revealed no significant difference between the C_3_ and C_4_ species examined (Fig. 6A, Supplementary Table S2). Although shorter BS cell length in C_4_ species compared to C_3_ species was observed (Fig. 6B, Supplementary Table S2), the calculated BS cell volumes were similar for the C_3_ and C_4_ species examined (Fig. 6C, Supplementary Table S2). Measurements from light micrographs of transverse leaf sections showed NAD-ME and NADP-ME species had the largest and smallest vein diameter, respectively, with C_3_ and PCK species being intermediate (Fig. 6D, Supplementary Table S2). As expected, leaf interveinal distance (IVD) was larger in C_3_ species than in C_4_ species (Fig. 6E, Supplementary Table S2), but among the C_4_ species the IVDs were not significantly different (Fig. 6E, Supplementary Table S2). Conversely, BS cell surface area per unit leaf area (*S*_b_) of C_4_ species was double that of C_3_ species (Fig. 6F, Supplementary Table S2).

## Discussion

Previous studies have shown that PD are more abundant at the M–BS cell interface in a C_4_ leaf compared to a C_3_ leaf (Botha, 1992; Danila *et al.*, 2016), presumably to accommodate the higher demand for metabolite transport between cell types in the C_4_ leaf that is required to support the C_4_ photosynthetic mechanism (Hatch and Osmond, 1976; Weber and von Caemmerer, 2010). However, little is known about the variation of PD density at the M–BS interface amongst C_4_ species and the different decarboxylation types. Modelling of metabolite movement between M and BS cells has been hampered by the lack of quantitative data on PD density at this key cellular interface (Danila *et al.*, 2016). To address this, we extended our PD density quantification to a larger subset of grasses representative of C_3_ photosynthesis, in both BEP (*n*=4) and PACMAD (*n*=1) clades, and in all the C_4_ decarboxylation types: NAD-ME (*n*=4), NADP-ME (*n*=6), and PCK (*n*=3) (Table 1). We confirmed that not only did all the C_4_ species examined have greater PD density than the C_3_ species, but that there was also substantial variation in how the high M–BS PD density was achieved among different C_4_ subtypes.

### Increased PD density in C_4_ grasses is a result of larger pit fields and/or more abundant PD per pit field area

Here we show that C_4_ species have evolved greater PD density than C_3_ species. Greater symplastic connectivity can be achieved by increasing the pit field area or by increasing the PD per pit field area. Interestingly we saw both solutions in our data, particularly in the M–BS interface. In NAD-ME species we saw an increase in pit field area without increasing PD per pit field area, while in PCK and NADP-ME species we saw an increase in both. We propose that the NAD-ME solution is due to their larger veins and thus their need for larger pit fields to facilitate transport. However, in PCK and NADP-ME types where veins are smaller it is sufficient just to increase PD per pit field area without much increase in pit field area to achieve the same effect. These results indicate that there is genetic plasticity in the way in which increased PD transport is achieved in C_4_ grasses.

### NAD-ME grasses have the greatest pit field area per M–BS interface among the C_4_ decarboxylation types

High PD density at the M–BS interface in NAD-ME grasses was solely determined by increases in pit field area per M–BS area driven by larger pit fields (rather than more numerous small pit fields). This is interesting in light of the unique aspects of NAD-ME BS cell anatomy (Dengler *et al.*, 1994). The distinct characteristics of BS cell chloroplasts and mitochondria in NAD-ME species compared to NADP-ME and PCK types (Dengler *et al.*, 1994) are consistent with the different solution they used to achieve high M–BS PD density. This supports the suggestion that PD function and formation are strongly coordinated with the function of both chloroplasts and mitochondria (Brunkard *et al.*, 2013; Wang *et al.*, 2017). Both the centripetal arrangement of mitochondria and larger BS cell cross-sectional area provide a longer diffusion pathlength in the NAD-ME leaf (von Caemmerer *et al.*, 2007). While this anatomical attribute minimises CO_2_ leakage across the M–BS interface, it may limit the rate of the C_4_ cycle activity (von Caemmerer *et al.*, 2007). More PD connections between M and BS cells allows rapid metabolite shuttling for the C_4_ cycle in the NAD-ME type (von Caemmerer *et al.*, 2007), therefore sustaining the high C_4_ photosynthetic rate (Henderson *et al.*, 1992; Pinto *et al.*, 2014). Correspondingly, the centrifugal arrangement of chloroplasts and mitochondria in the NADP-ME type presents a greater possibility of CO_2_ leakage (von Caemmerer *et al.*, 2007). However, fewer PD between M and BS cells in NADP-ME leaves, in combination with the suberin lamella surrounding the BS, minimises this possibility (von Caemmerer *et al.*, 2007). It is interesting that there was almost as much diversity in the PD density at the M–BS interface among PCK species as there was across C_4_ grasses as a whole. This wide range of PD densities in the PCK species examined could result from both PCK and NAD decarboxylation located in the BS (von Caemmerer and Furbank, 2003) together with their considerable variation in BS cell cross-sectional areas, BS chloroplast morphology and positioning, and abundance of mitochondria in the BS (Hattersley and Browning, 1981).

### Large PD size between photosynthetic cells is found in leaves of all the grass species examined

Using TEM sections for PD studies presents advantages and disadvantages, highly dependent on the purpose of the study (i.e. ultrastructure versus quantification). For our purpose of analysing many samples, SEM analysis was a more rapid and rigorous way to quantify the cross-sectional area of individual PD in grasses. Random tearing of leaf tissue to reveal M–BS or M–M cell interfaces allowed us to expose the cross-sectional area of PD, almost always within the middle cavity because the tissue tended to separate along the middle lamella between tissue layers. In our previous paper (Danila *et al.*, 2016), our PD area measurements from TEM micrographs used only PD cross-sections with distinct central desmotubules. Similar measurements taken from previously published TEM micrographs of grass leaf PD (fig. 8 from Botha, 1992) generated values similar to both our TEM and SEM results. In fact, the similarity of PD area values we obtained when we compared our TEM and SEM measurements for *O. sativa*, *Z. mays*, and *S. viridis* encouraged us to use SEM in place of TEM for PD area measurement. We find it interesting that the PD areas we obtained in all cases were very large (about 0.006 μm^2^), the diameter being in the range of 90 nm, while the majority of published values for land-plant PD, many of which were obtained from root PD measurements, have a diameter of 50 nm or smaller (Overall, 1999; Ehlers and Kollmann, 2001).

### Anatomical enablers of C_4_ photosynthesis

It has been proposed that enlargement of the BS cells in C_4_ leaves compared to C_3_ leaves and their ‘functionalisation’ by increases in chloroplast number was an early step in C_4_ evolution (Sage, 2004). Clearly, in this study we showed that there is little evidence for BS cells in C_4_ grasses being larger in volume than their C_3_ counterparts. What we saw instead was a distinctively large BS surface area to leaf area ratio (*S*_b_) in C_4_ grass leaves compared to C_3_ leaves, consistent with the report of Hattersley (1984). We therefore argue that increasing *S*_b_, but not BS cell size, is important in C_4_ leaf physiology (von Caemmerer *et al.*, 2007). This finding emphasises the importance of looking at the 3-D geometry of cells in addition to the 2-D view for a more global cell perspective and for improved accuracy in terms of reporting measured values (Théroux-Rancourt *et al.*, 2017). Large IVD is also not always a clear indication of C_3_ anatomy because it can be interpreted as either an increase in interveinal M cell number (as in C_3_ grasses) or an increase in BS cell cross-sectional area (as in most NAD-ME and PCK grasses). Our observations showed consistently greater PD density on the M–BS interface in all the C_4_ species examined relative to the C_3_ species. Another potentially useful diagnostic character is pit field density, as seen in the substantial difference between the NAD-ME types and other decarboxylation types. This could be used to distinguish not only C_3_ from C_4_ photosynthesis but also among C_4_ biochemical types, at least in grasses.

### PD density, metabolite flux, and CO_2_ diffusion in the C_4_ BS

PD density at the M–BS interface affects not only metabolite diffusion but also leakage of inorganic carbon and O_2_ out of the BS compartment, an important determinant of the efficiency of C_4_ photosynthesis (von Caemmerer and Furbank, 2003). While it is difficult to directly determine the CO_2_ concentration in the BS of C_4_ plants, this parameter can be modelled using certain assumptions concerning leaf cell anatomical dimensions, inorganic carbon equilibration, and diffusion properties of membranes and PD (Furbank and Hatch, 1987; Jenkins *et al.*, 1989; von Caemmerer and Furbank, 2003). Based on permeability coefficients determined for metabolites moving into isolated BS cells through PD (Weiner *et al.*, 1988) and for CO_2_ in C_4_ leaves and isolated BS cells (Furbank *et al.*, 1989; Jenkins *et al.*, 1989), Jenkins *et al.* (1989) calculated that approximately 40% of the CO_2_ leakage from the BS occurs via an apoplastic route and 60% via the PD. For oxygen, which moves poorly through lipid bilayers and polymeric barriers such as suberin (Jenkins *et al.*, 1989), the majority of diffusion will be through the aqueous route, i.e. via PD. Likewise, bicarbonate moves poorly through lipid membranes and will diffuse out of the BS mostly via PD (Furbank *et al.*, 1989). Given these modelled values for inorganic carbon and O_2_ movement at the M–BS interface, the ‘optimal’ PD density for a C_4_ leaf would be a compromise between high density for metabolite transport and dissipation of O_2_ (in species with PSII in the BS chloroplast), and leakage of inorganic carbon from the BS compartment. The quantification of PD density made here is a first step towards enabling more accurate modelling of metabolite flux and leakage of inorganic carbon from the BS across a range of species and decarboxylation types (Wang *et al.*, 2014). The key parameter required for predicting metabolite gradients and leaf cell metabolite concentrations required to support the C_4_ pathway is the proportion of M–BS interface comprised of PD pores (Osmond, 1971; Hatch and Osmond, 1976). The current work provides these data for a range of species. The values provide an upper limit given that not all PD may be functional and the obstruction of the PD area by the desmotubule has not been taken into account. Nevertheless, these data facilitate modelling of inorganic carbon leakage rates from the BS without resorting to the use of permeability values obtained from isolated cells (Jenkins *et al.*, 1989). This will be particularly useful for interspecific comparisons given the diversity in suberisation of the M–BS interface between species and the diverse arrangements of organelles and cellular sites of C_4_ acid decarboxylation across the three biochemical types.

### Evolution of symplastic connections to the BS in C_4_ leaves

It has been reported previously that the transition from C_3_ to C_4_ photosynthesis involved a series of genetic alterations, mostly gain of function, leading to numerous changes in plant anatomy and biochemistry (Sage *et al.*, 2012; Bräutigam *et al.*, 2014; Wang *et al.*, 2014; Emms *et al.*, 2016). Recently it was suggested that evolution of C_4_ photosynthesis has involved a change in the apoplastic transport of sugars in the BS cells of C_4_ leaves (Emms *et al.*, 2016). A sugar effluxer (or SWEET protein) appears to have been recruited from a relatively minor role in the M cells of C_3_ grasses to become one of the most highly expressed transcripts in the C_4_ BS cells (Emms *et al.*, 2016). While we have not as yet identified the genetic changes responsible, we show here that PD density was at least doubled in C_4_ species compared to C_3_ species, a clear indication of enhanced expression of PD developmental genes. However, to date, the genes underpinning PD development remain largely unknown (Brunkard and Zambryski, 2017). It is intriguing to consider whether the proposed evolutionary pressures to recruit a highly expressed sugar effluxer to the BS cells of C_4_ plants were linked to the proliferation of PD at the M–BS interface in C_4_ leaves. Recent data from genome-wide gene-tree/species-tree reconciliation in grasses has revealed multiple functional categories of genes, of which 10 genes were found to have plausible association with the symplastic transport function (Emms *et al.*, 2016). This leads us to be optimistic that the discovery of candidate genes controlling PD development may be not too far away.

## Supplementary data

Supplementary data are available at *JXB* online.

Dataset S1. Single-copy orthologous gene sequences used for phylogenetic tree construction (supplied as a BZIP2 file).

Fig. S1. Light micrographs of transverse sections of C_3_ and C_4_ grass leaves.

Table S1. Quantitative plasmodesmata traits of the 18 grass species examined.

Table S2. Leaf anatomical traits quantified in the 18 grass species examined.

Supplementary Figure S1 and Tables S1_S2

Dataset S1
