# Supplementary material for: Multiple mechanisms for enhanced plasmodesmata density in disparate subtypes of C4 grasses
Source: J Exp Bot. 2018 Jan 2;69(5):1135–45. doi: 10.1093/jxb/erx456 (PMC6018992; doi:10.1093/jxb/erx456)
Supplement: Supplementary Figure S1 and Tables S1_S2 [file erx456_suppl_supplementary-figure-s1-table-s1_s2.pdf]

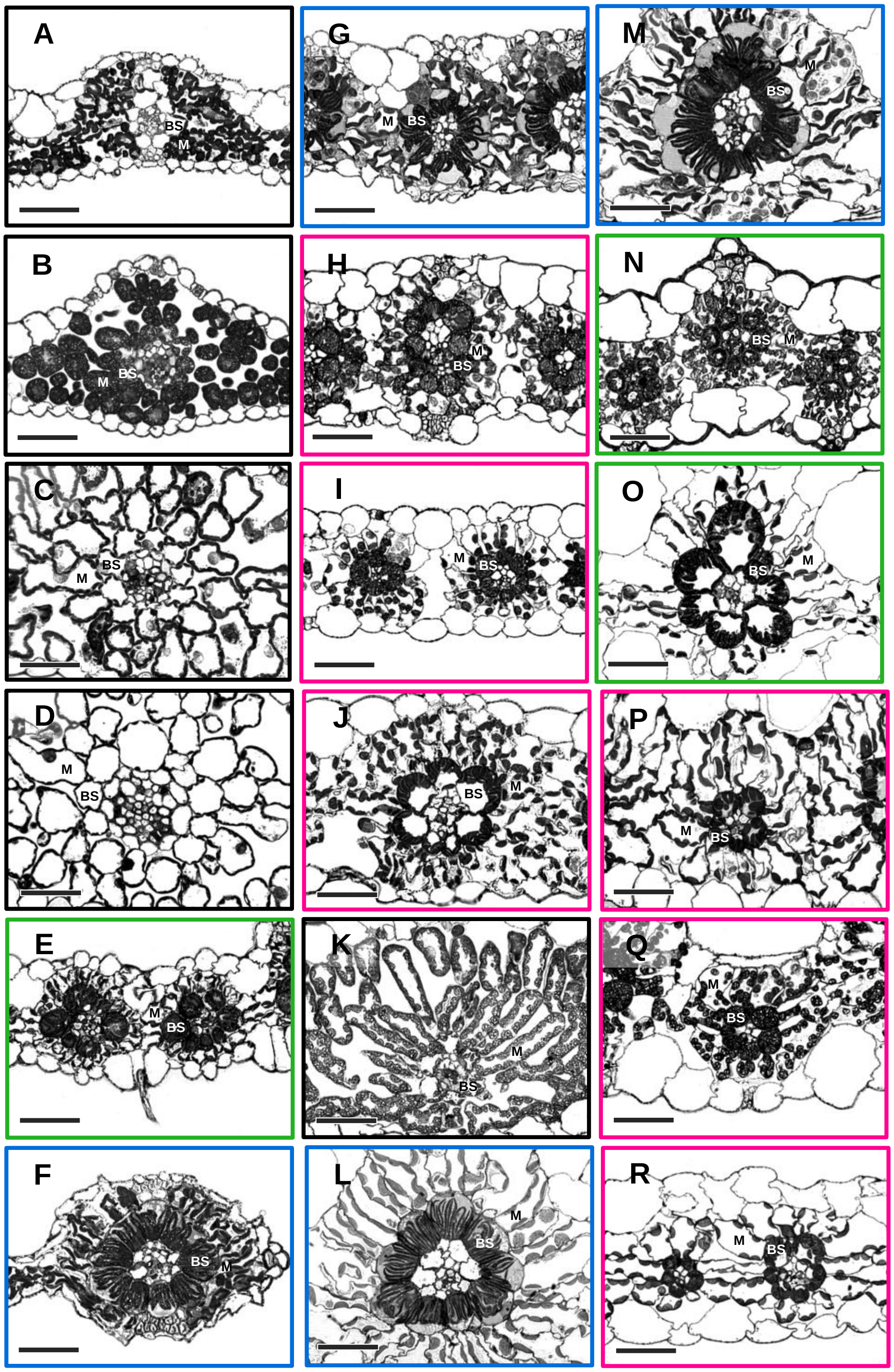

Figure S1 Light micrographs of transverse sections of C_3 and C_4 grass leaves. (A-D) C_3 BEP species, black frame: (A) *Oryza sativa*, (B) *Brachypodium distachyon*, (C) *Hordeum vulgare*, and (D) *Triticum aestivum.* (E, N-O) C_4 PCK, green frame: (E) *Chloris gayana*, (N) *Panicum maximum*, and (O) *Urochloa panicoides*. (F-G, L-M) C_4 NAD-ME, blue frame: (F) *Leptochloa fusca*, (G) *Astrebla lappacea*, (L) *Panicum coloratum*, and (M) *Panicum miliaceum*. (H-J, P-R) C_4 NADP-ME, magenta frame: (H) *Paspalum dilatatum*, (I) *Sorghum bicolor*, (J) *Zea mays*, (P) *Setaria viridis*, (Q) *Cenchrus ciliaris*, and (R) *Panicum antidotale*. (K) C_3 PACMAD, black frame: *Panicum bisulcatum*. Mesophyll (M) and bundle sheath (BS) cells are labelled. Bars = 50 μm.

Table S1. Quantitative plasmodesmata traits of the 18 grass species examined.

Grass species	Photosynthetic type	M-BS PD per μm^2 pit field	M-BS pit field area per cell interface area (%)	M-BS PD per μm^2 cell interface	M-M PD per μm^2 pit field	M-M pit field area per cell interface area (%)	M-M PD per μm^2 cell interface	Pit field area (μm^2)	PD area ($x10^{-3}$ μm^2)	PD area per interface area ($x10^{-3}$ μm^2 μm^{-2})	PD area per leaf area ($x10^{-3}$ m^2 m^{-2})
O. sativa	C_3, BEP	35 ± 0.4*	2.8 ± 0.07*	1.0 ± 0.02*	39 ± 0.4*	4.1 ± 0.15*	1.6 ± 0.08*	0.3 ± 0.06	6.1 ± 0.20	6.0 ± 0.07	6.6 ± 0.07
B. distachyon	C_3, BEP	40 ± 0.6	2.8 ± 0.15	1.1 ± 0.06	41 ± 0.4	3.4 ± 0.17	1.4 ± 0.07	0.4 ± 0.03	11.3 ± 0.33	12.9 ± 0.23	11.1 ± 0.15
H. vulgare	C_3, BEP	39 ± 0.6	6.2 ± 0.17	2.4 ± 0.06	42 ± 0.6	7.0 ± 0.08	2.9 ± 0.04	0.4 ± 0.03	9.8 ± 0.29	23.6 ± 0.27	21.1 ± 0.29
T. aestivum	C_3, BEP	47 ± 1.0*	5.5 ± 0.32*	2.6 ± 0.15*	41 ± 0.7*	3.7 ± 0.22*	1.5 ± 0.09*	1.6 ± 0.17	9.3 ± 0.30	24.0 ± 0.46	28.2 ± 0.71
C. gayana	C_4 PCK, PACMAD	58 ± 1.2	14.8 ± 0.28	8.6 ± 0.17	62 ± 1.2	10.7 ± 0.34	6.6 ± 0.21	0.3 ± 0.04	5.7 ± 0.20	48.5 ± 0.56	113.7 ± 1.02
L. fusca	C_4 NAD-ME, PACMAD	41 ± 0.5	20.5 ± 0.45	8.3 ± 0.18	67 ± 0.4	8.7 ± 0.41	5.8 ± 0.28	1.5 ± 0.40	7.1 ± 0.33	58.9 ± 0.87	111.1 ± 1.58
A. lappacea	C_4 NAD-ME, PACMAD	57 ± 0.7	21.5 ± 0.20	12.2 ± 0.12	58 ± 0.9	9.5 ± 0.27	5.5 ± 0.16	1.4 ± 0.17	5.5 ± 0.16	67.7 ± 0.61	165.2 ± 3.18
P. dilatatum	C_4 NADP-ME, PACMAD	53 ± 2.9	12.4 ± 0.25	6.6 ± 0.13	49 ± 1.0	5.3 ± 0.18	2.6 ± 0.09	0.9 ± 0.14	7.2 ± 0.18	47.3 ± 0.43	94.6 ± 0.91
S. bicolor	C_4 NADP-ME, PACMAD	66 ± 1.0	8.9 ± 0.26	5.9 ± 0.18	64 ± 1.6	6.4 ± 0.25	4.1 ± 0.16	0.4 ± 0.05	7.8 ± 0.17	45.9 ± 0.49	70.7 ± 0.58
Z. mays	C_4 NADP-ME, PACMAD	66 ± 0.7*	11.4 ± 0.27*	7.5 ± 0.18*	78 ± 0.6*	14.4 ± 0.24*	11.2 ± 0.18*	0.7 ± 0.08	7.3 ± 0.12	55.2 ± 0.46	109.0 ± 0.86
P. bisulcatum	C_3, PACMAD	56 ± 0.6	6.7 ± 0.33	3.7 ± 0.19	52 ± 0.4	3.6 ± 0.09	1.9 ± 0.05	0.5 ± 0.04	7.0 ± 0.14	26.3 ± 0.40	19.5 ± 0.17
P. coloratum	C_4 NAD-ME, PACMAD	46 ± 0.4	25.7 ± 0.41	11.9 ± 0.19	50 ± 0.7	10.1 ± 0.42	5.0 ± 0.21	1.2 ± 0.10	7.9 ± 0.24	94.5 ± 0.92	155.3 ± 2.59
P. miliaceum	C_4 NAD-ME, PACMAD	52 ± 0.7	19.3 ± 0.29	10.0 ± 0.15	59 ± 0.4	5.8 ± 0.27	3.4 ± 0.16	2.4 ± 0.40	7.9 ± 0.38	78.8 ± 1.15	121.6 ± 1.14
P. maximum	C_4 PCK, PACMAD	54 ± 0.3	9.2 ± 0.27	5.0 ± 0.15	54 ± 0.3	5.8 ± 0.17	3.1 ± 0.09	0.6 ± 0.05	6.4 ± 0.22	31.5 ± 0.42	62.6 ± 0.55
U. panicoides	C_4 PCK, PACMAD	72 ± 0.6	15.2 ± 0.30	10.9 ± 0.21	72 ± 0.6	3.7 ± 0.06	2.6 ± 0.04	0.7 ± 0.05	6.4 ± 0.16	70.2 ± 0.64	142.0 ± 0.75
S. viridis	C_4 NADP-ME, PACMAD	73 ± 0.9*	12.7 ± 0.34*	9.3 ± 0.25*	75 ± 0.8*	8.5 ± 0.14*	6.4 ± 0.11*	0.6 ± 0.07	6.8 ± 0.16	63.0 ± 0.64	102.6 ± 0.70
C. ciliaris	C_4 NADP-ME, PACMAD	72 ± 1.0	9.5 ± 0.16	6.8 ± 0.12	72 ± 1.0	6.3 ± 0.13	4.5 ± 0.09	0.6 ± 0.06	6.9 ± 0.19	47.2 ± 0.45	69.6 ± 0.76
P. antidotale	C_4 NADP-ME, PACMAD	59 ± 0.5	13.0 ± 0.18	7.7 ± 0.11	59 ± 0.5	5.0 ± 0.11	2.9 ± 0.07	0.7 ± 0.06	7.2 ± 0.14	55.6 ± 0.39	91.4 ± 0.50

Asterisks indicate values previously reported in (Danila et al., 2016). M-BS = mesophyll-bundle sheath, PD = plasmodesmata, M-M = mesophyll-mesophyll, PD area = cross sectional area of individual PD measured from SEM.

Table S2. Leaf anatomical traits quantified in the 18 grass species examined.

Grass species	Photosynthetic type	BS cell area (μm^2)[a]	BS cell length (μm)[a]	BS cell volume (μm^3)[a]	Vein diameter (μm)[b]	IVD (μm)[b]	S_b (m^2 m^{-2})[b]
O. sativa	C_3, BEP	180 ± 6.2	51 ± 1.8	9113 ± 130.3	53 ± 3.6	189 ± 3.1	1.1 ± 0.06*
B. distachyon	C_3, BEP	340 ± 15.7	50 ± 2.0	16927 ± 300.0	61 ± 1.7	322 ± 7.2	0.9 ± 0.05
H. vulgare	C_3, BEP	349 ± 11.4	88 ± 2.8	30609 ± 401.6	67 ± 3.1	381 ± 12.3	0.9 ± 0.07
T. aestivum	C_3, BEP	630 ± 47.4	130 ± 9.1	81758 ± 2433.3	119 ± 2.9	353 ± 2.0	1.2 ± 0.17*
C. gayana	C_4 PCK, PACMAD	618 ± 31.5	32 ± 0.7	19767 ± 313.2	61 ± 5.8	99 ± 6.2	2.3 ± 0.09
L. fusca	C_4 NAD-ME, PACMAD	756 ± 40.7	41 ± 1.0	30648 ± 525.8	80 ± 3.0	143 ± 16.9	1.9 ± 0.14
A. lappacea	C_4 NAD-ME, PACMAD	570 ± 26.1	27 ± 0.8	16883 ± 258.3	93 ± 4.0	109 ± 0.5	2.4 ± 0.28
P. dilatatum	C_4 NADP-ME, PACMAD	241 ± 13.5	36 ± 1.2	8743 ± 165.4	50 ± 2.2	103 ± 4.4	2.0 ± 0.10
S. bicolor	C_4 NADP-ME, PACMAD	360 ± 19.7	29 ± 0.6	10474 ± 177.8	43 ± 1.2	102 ± 4.5	1.5 ± 0.05
Z. mays	C_4 NADP-ME, PACMAD	586 ± 44.6	42 ± 1.1	24318 ± 566.5	85 ± 4.6	155 ± 5.1	2.0 ± 0.08*
P. bisulcatum	C_3, PACMAD	188 ± 9.5	63 ± 2.1	11778 ± 205.3	51 ± 2.0	274 ± 5.5	0.7 ± 0.01
P. coloratum	C_4 NAD-ME, PACMAD	463 ± 20.6	27 ± 0.5	12421 ± 174.2	110 ± 2.5	234 ± 14.4	1.6 ± 0.16
P. miliaceum	C_4 NAD-ME, PACMAD	816 ± 28.0	23 ± 0.6	18484 ± 226.9	129 ± 3.7	263 ± 6.8	1.5 ± 0.05
P. maximum	C_4 PCK, PACMAD	293 ± 22.1	54 ± 1.8	15699 ± 373.3	47 ± 1.5	89 ± 2.7	2.0 ± 0.06
U. panicoides	C_4 PCK, PACMAD	1175 ± 87.2	44 ± 1.1	51545 ± 1167.1	99 ± 3.9	185 ± 6.7	2.0 ± 0.02
S. viridis	C_4 NADP-ME, PACMAD	137 ± 6.4	33 ± 1.0	4528 ± 73.0	49 ± 1.7	126 ± 2.6	1.6 ± 0.04*
C. ciliaris	C_4 NADP-ME, PACMAD	218 ± 9.5	50 ± 1.3	10913 ± 159.3	60 ± 3.7	157 ± 21.2	1.5 ± 0.09
P. antidotale	C_4 NADP-ME, PACMAD	225 ± 6.3	47 ± 1.2	10634 ± 116.9	44 ± 2.4	102 ± 6.6	1.6 ± 0.04

[a]Measured from confocal micrographs derived from whole leaf z-stacks.
[b]Measured from light micrographs of transverse leaf sections.
Asterisks indicate values previously reported in (Danila et al., 2016). BS = bundle sheath, VB = vascular bundle, IVD = interveinal distance, S_b = bundle sheath surface area per unit leaf area.
